# The association of stroke severity with health-related quality of life in survivors of acute cerebrovascular disease and their informal caregivers during the first year post stroke: a survey study

**DOI:** 10.1007/s11136-020-02516-3

**Published:** 2020-05-09

**Authors:** Angela S. Labberton, Liv Ariane Augestad, Bente Thommessen, Mathias Barra

**Affiliations:** 1 Health Services Research Unit, Akershus University Hospital, PO Box 1000, 1478 Lørenskog, Norway; 2 Department of Health Management and Health Economics, Medical Faculty, University of Oslo, Oslo, Norway; 3 Department of Neurology, Akershus University Hospital, Lørenskog, Norway

**Keywords:** Informal caregivers, Cerebrovascular disease, Stroke, Transient ischaemic attack, Quality of life

## Abstract

**Purpose:**

To describe the health-related quality of life (HRQoL) of caregivers and survivors of transient ischaemic attack (TIA) and stroke during one year post discharge in comparison to age- and sex-matched population norms; and to analyse the association of initial stroke severity, measured by a routinely used stroke-specific scale, on subsequent HRQoL of caregivers and survivors.

**Methods:**

Cohort of hospitalized patients with TIA and stroke discharged alive from a large university hospital in Norway, and their informal caregivers. Questionnaires at 3 and 12 months post discharge were filled out by caregivers (*n* = 320 and *n* = 326, respectively) and survivors (*n* = 368 and *n* = 383, respectively). Multivariable linear regression analyses tested associations between initial stroke severity (National Institutes of Health Stroke Scale, NIHSS) and HRQoL (EQ-5D-3L) in caregivers and survivors.

**Results:**

Caregivers of survivors with TIA or stroke did not report lower HRQoL than matched norms. There was some evidence of an association of the NIHSS with caregiver HRQoL at 3 months only (age–sex-adjusted coefficient − 0.01, *p* = 0.008), however, this was attenuated after additional adjustments. Survivors with stroke, but not TIA, reported lower HRQoL than population norms at both time points. There was a negative association between higher NIHSS scores and survivors’ HRQoL; fully adjusted coefficient − 0.01 at both time points (*p* = 0.001).

**Conclusion:**

The informal caregivers and survivors with TIA did not report lower than expected HRQoL. Increasing stroke severity was associated with decreasing HRQoL among survivors, but had limited predictive value among caregivers. Other factors may therefore be better indicators of ‘at risk’ caregivers.

**Electronic supplementary material:**

The online version of this article (10.1007/s11136-020-02516-3) contains supplementary material, which is available to authorized users.

## Introduction

Stroke is one of the leading causes of death and disability worldwide [1]. The survivors of stroke often have complex persisting impairments requiring long-term care. With increased focus on earlier discharge and re-integration of patients into the community, the role of family and other informal caregivers is becoming more important. Accordingly, there are a growing number of studies addressing the quality of life (QoL) of informal caregivers of stroke survivors [2–19]. While QoL is a broad assessment of an individual’s subjective well-being or life satisfaction, this study investigates the narrower concept of health-related quality of life (HRQoL). Specifically, in this paper HRQoL refers to the value of different health states that are commonly integrated over time to estimate quality adjusted life-years (QALYs) allowing cost-effectiveness analyses [20].

A common finding among informal caregivers of stroke survivors is a decrease in HRQoL compared to norm values or controls [7, 8, 13, 17, 21, 22]. Decreased HRQoL has been shown to be associated with increasing caregiver age [2, 4, 5, 9, 10, 14], caregiver burden [2, 3, 18, 22], anxiety or depression in the caregiver [9, 16–18] or survivor [3, 4, 6, 9, 14, 18], and lower levels of function in the survivor [2–4, 11, 14, 17–19].

Acute cerebrovascular disease (CVD) results in a large variation of outcomes, ranging from full recovery to severe disability or death. It is plausible that increasing disease severity in the survivor has an impact on caregiver HRQoL. Transient ischaemic attack (TIA), which by definition does not leave persisting neurological deficits beyond 24 h, represents the mildest form of symptomatic CVD. Still, survivors of TIA and minor stroke do report subsequent problems with cognitive impairment, depression, and fatigue [23–25]. To our knowledge, caregiver HRQoL in cases of TIA has not been reported previously.

The National Institutes of Health Stroke Scale (NIHSS) is the most widely used and validated scale for neurological impairment among stroke patients [26–28]. It is a routine part of the initial stroke assessment on admission to hospital. Hence, if NIHSS scores predict subsequent HRQoL in stroke survivors and caregivers, this can be incorporated into discharge planning or risk scoring models.

Existing studies of caregivers’ HRQoL that do include measures of impairment in the stroke survivors predominantly use generic disability scales such as the modified Rankin Scale (mRS) [14], or measures of ability to perform activities of daily living (ADL) such as the Barthel Index (BI) [4, 5, 9, 11, 15, 17–19, 21, 22] or the Functional Independence Measure (FIM) [6]. While these instruments provide valuable information regarding the survivors’ level of function, they may not detect stroke-specific impairments, and may not be measured in all patients in routine clinical practice.

The aim of this study was to describe the HRQoL of caregivers and survivors with TIA and stroke during the year following the acute hospital admission, and to measure the association between initial stroke severity, as measured by the NIHSS, and subsequent HRQoL in both caregivers and survivors. We hypothesized that the TIA survivors and their caregivers would not have significantly different HRQoL to age- and sex-matched population norms, and that HRQoL in survivors and caregivers would display a gradient along increasing stroke severity.

## Methods

### Study setting and participants

This study used data from the Norwegian Stroke—Paths of Treatment (NOR-SPOT) cohort, collected at Akershus University Hospital between 15th February 2012 and 15th March 2013 to investigate health service delivery and outcomes for stroke patients. All consecutive admissions to the stroke unit, plus the few additional stroke patients admitted elsewhere due to overcrowding, were prospectively included. The hospital is located in greater metropolitan Oslo and has about 500,000 inhabitants in its catchment area (~ 10% of Norway’s population). Both urban and rural areas are represented, and there is no self-selection for acute admissions in Norway.

**Fig. 1:**
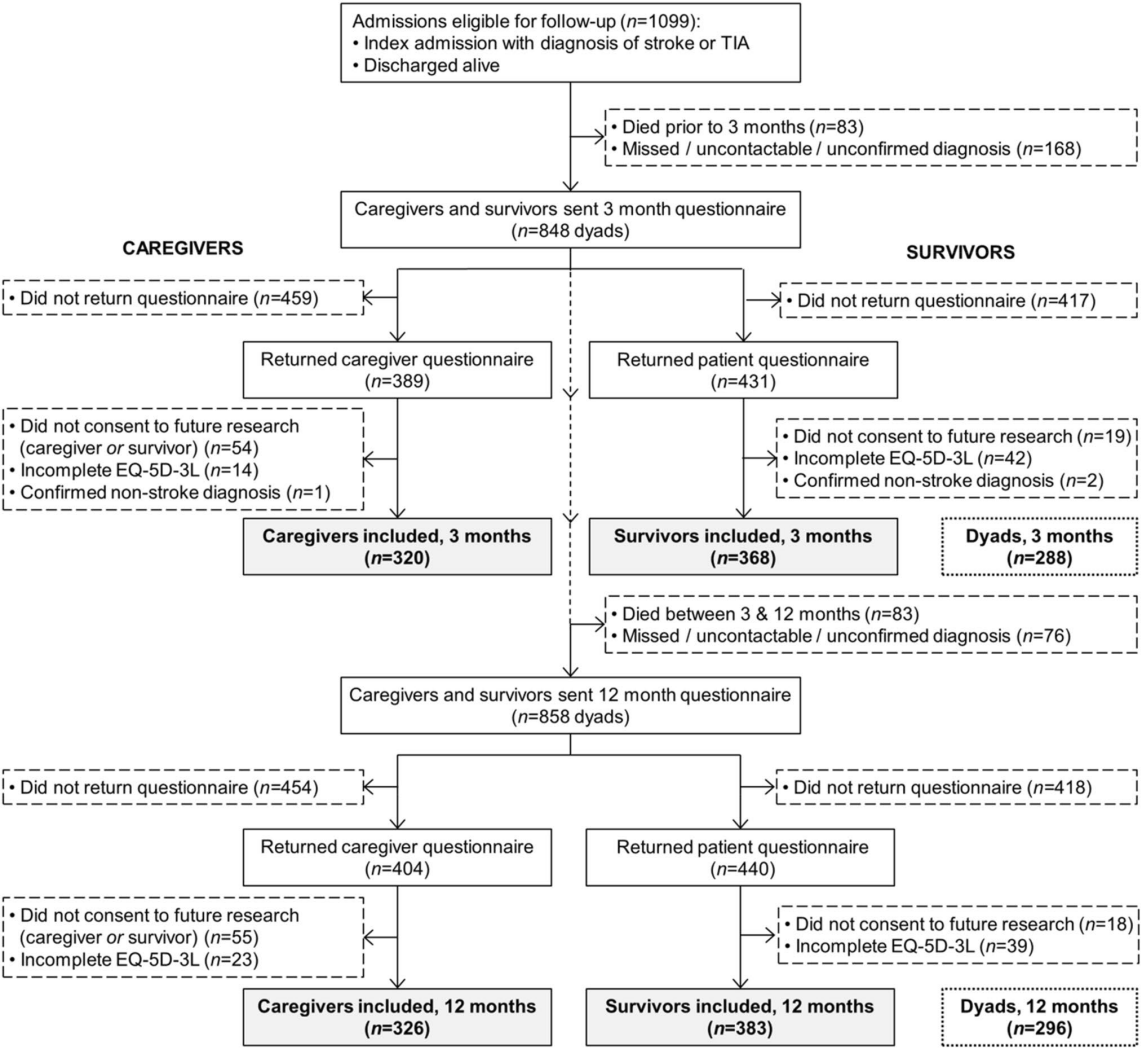
Inclusion flowchart. *TIA* transient ischaemic attack

All patients discharged alive with an acute CVD diagnosis received postal questionnaires after 3 and 12 months from acute hospital discharge. CVD diagnoses were defined by International Classification of Diseases-10th revision (ICD-10) codes: ischaemic stroke (I63.X); intracerebral haemorrhage (ICH) (I61.X); TIA (G45.X, excluding G45.4). In cases of multiple admissions with acute CVD, only the first admission during the data collection period was followed up. There were two separate questionnaires at each follow-up time point; one each for the survivor and caregiver. The survivors were instructed to give the caregiver form to their partner or spouse to fill out if they had one, or to the first relative or close friend they met if not. Survivors and caregivers who returned the questionnaires and provided written consent to participation were included in this study; see Fig. 1 for the inclusion flowchart.

### Variable definitions

Health-related quality of life was measured in both survivors and caregivers using the generic EQ-5D-3L instrument [29], licensed by the EuroQol Group. The EQ-5D-3L is a generic instrument to measure HRQoL, developed for broad application and cross-disease comparison. The EQ-5D-3L has been shown to be valid for stroke [20, 30], and is suitable for use on both the survivors and on their informal caregivers [20]. Furthermore, the EQ-5D-3L is accompanied by value sets assigning an associated health state value—i.e. HRQoL—to each of the health states described by the instrument. Population norms for Norway have been published [31], allowing comparison with the general population.

The EQ-5D-3L consists of a descriptive system with five dimensions and a visual analogue scale (VAS). The five dimensions (mobility, self-care, usual activities, pain/discomfort, and anxiety/depression) are each rated with three possible levels: 1 = ‘no problems’; 2 = ‘some problems’; and 3 = ‘extreme problems’. The descriptive system therefore contains ${3}^{5}= 243$ distinct health states. We used the UK tariff [32], which is the most commonly used tariff in Norway [31], and which was also used to estimate the Norwegian population norm values [31]. The health state values are anchored at 1 (‘full health’), while the health state value 0 marks the border between health states ‘better than’ versus ‘worse than’ immediate death [32].

The caregiver questionnaires collected data on how often the caregivers provided different types of help to the survivor during the preceding seven days, and whether the caregiver had to take time off from paid employment for caregiving-related reasons. Help provided included the following basic ADLs: dressing, eating, self-care; and the following instrumental ADLs: chores, cooking, transport, contact with health service providers, accompanying to health care appointments. Caregivers indicated the amount of help by marking one of the following options: ‘no’, ‘at least once’, ‘almost daily’, ‘several times per day’, or ‘unsure’. Caregivers’ sociodemographic data (age, sex, education, and relationship to the survivor) were also collected.

Data regarding the type and severity of the stroke and discharge destination of the survivor were collected during the acute hospital admission. Stroke severity was determined by the NIHSS score within 24 h of admission by the neurologist on duty. Where a prospective NIHSS score was unavailable, patients were scored retrospectively by the first author using admission records and a validated algorithm [33]. The proportions of NIHSS scores assigned retrospectively were 16.6% (43/259) and 19.7% (53/269) among the survivors with a stroke diagnosis included at 3 months and 12 months, respectively. The NIHSS is an 11-item neurological examination designed specifically for stroke, and is reliable and valid [34, 35]. Scores range from 0 to 42 with lower scores indicating milder strokes. Stroke severity was categorized according to NIHSS scores as ‘mild’ (NIHSS ≤ 3), ‘moderate’ (NIHSS 4–10), and ‘severe’ (NIHSS > 10) [36, 37]. Discharge destination was classified as ‘home or inpatient rehabilitation’ vs. ‘nursing home’ vs. ‘other’ (e.g. hospital transfer).

Finally, self-rated Hospital Anxiety and Depression Scale (HADS) [38] scores were collected in caregivers and survivors, and global function (measured with the mRS [39]) in the survivors. For the HADS, we considered a score of ≥ 8 on either the anxiety or depression sub-scale to indicate an anxiety or depression mood disorder, respectively [40].

### Statistical analysis

Continuous variables are presented as mean and standard deviation (SD), or median and interquartile range (IQR), and categorical variables as frequency and percentage. The magnitude of deviation of participants’ EQ-5D-3L health state values from the age- and sex-matched norm value [31] were calculated as ${\Delta }_{U,i}={U}_{i}-{U}_{n\left(i\right)}$, where ${U}_{i}$ is the health state value of individual $i$, and ${U}_{n\left(i\right)}$ denotes the age- and sex-matched expected health state value (according to the population norm from Stavem et al. [31]). Independent *t* tests were used for significance testing of the deviation from matched norm scores. The change in ${\Delta }_{U}$ at 3 versus 12 months was assessed by the *t* test for partially overlapping samples (R package Partiallyoverlapping [41]). For the stroke survivors, who may have persisting problems, and their caregivers, the EQ-5D-3L dimensions were also analysed separately, and changes over time in the proportion experiencing problems with each dimension were assessed with the *z*-test for partially overlapping samples (R package Partiallyoverlapping [41]).

To further examine the association of stroke severity with EQ-5D-3L health state values, we performed linear regressions on the deviation of health state values from the population norm (${\Delta }_{U}$) at each time point. For survivors with TIA a NIHSS score of zero was imputed for these analyses, since TIA by definition does not result in persisting neurological impairments. The initial model (Model 1) included only NIHSS scores as explanatory variable; the second model (Model 2) was also adjusted for age and sex; and the final model (Model 3) was additionally adjusted for discharge diagnosis (TIA, ischaemic stroke, ICH), discharge destination, EQ-5D-3L state value of the opposite member of the caregiver–survivor dyad. Model 3 was therefore restricted to dyads responding at 3 months and 12 months, respectively. Finally, as a sensitivity analysis, Model 4 additionally included variables with incomplete data, but which are known to be important determinants of caregiver HRQoL: caregiver and survivor HADS scores, and survivor mRS scores.

To assess selection bias, we compared descriptive characteristics of the survivors with participating and non-participating caregivers, and of participating and non-participating survivors, with *t* tests, Wilcoxon rank-sum test, or chi-squared tests. Non-participating individuals were defined as those who were eligible for follow-up at each time point (i.e. discharge diagnosis of stroke or TIA, and alive at follow-up time point), but who were not included in our analyses for any reason (e.g. did not return questionnaire, no consent, incomplete EQ-5D-3L). Because our EQ-5D-3L data were collected via a postal survey, a sensitivity analysis was also performed comparing caregiver health state values to web-based and postal survey population norm values separately.

Independent variables included in the regression models were assessed for multicollinearity using the variation inflation factor, and found to be acceptable. Missing values were excluded from analyses and all tests were two-tailed with significance level 0.05. Analyses were performed using the statistical software StataIC (StataCorp, College Station, TX) or R (R Foundation for Statistical Computing, Vienna, Austria).

### Ethics

Collection of data for the NOR-SPOT project was considered to be quality assurance by the Regional Committee for Medical and Health Research Ethics (REC), and approval was granted by the Data Protection Officer at Akershus University Hospital (ref.no. 11-076). The return of the follow-up questionnaires was voluntary for both survivors and caregivers. Ethical approval for the study of the caregivers’ HRQoL was granted by the Regional Committee for Medical and Health Research Ethics under a separate application (ref.no. 2015/1820) on the condition that both the caregiver and survivor had consented to participate in future research. In cases where the survivor was unable to consent themselves, their proxy gave consent on their behalf. Written, informed consent was obtained from all individual participants included in the current study.

## Results

In all, 320 caregivers were included at 3 months and 326 at 12 months. The respective numbers for survivors were 368 and 383 (Fig. 1). Of the 368 survivors included at 3 months, 21 (5.7%) died before the 12-month follow-up. The caregivers’ mean age was 63 years and just under two-thirds were female (Table 1). The majority were spouses (62% at 3 months; 65% at 12 months), and around 40% were currently working. The survivors had mean age 72 and 71 years at 3 and 12 months, respectively, around 40% were females, and the majority had mild, ischaemic strokes (median NIHSS 3; 61% ischaemic stroke). Help with completing the EQ-5D-3L was given to 22.6% of survivors at 3 months, and 19.8% at 12 months.

**Table 1:** Caregiver and survivor characteristics

	Participants, 3 months	Missing values	Participants, 12 months	Missing values
**Caregivers**, *n*	320		326	
Age in years, mean (SD)	62.5 (13.3)	16	62.8 (12.9)	18
Female sex	193 (60.3)	21	208 (63.8)	25
Relationship to survivor		1		1
Spouse/partner	198 (61.9)		212 (65.0)	
Adult child	101 (31.6)		88 (27.0)	
Other	20 (6.3)		25 (7.7)	
Highest education		13		12
Primary school	69 (21.6)		59 (18.1)	
Secondary school	129 (40.3)		129 (39.6)	
University	109 (34.1)		126 (38.7)	
Currently working	120 (37.5)	22	135 (41.4)	14
HADS anxiety score		13		13
Mean (SD)	4.9 (4.0)		5.1 (4.2)	
Score ≥ 8	71 (23.1)		78 (24.9)	
HADS depression score		12		13
Mean (SD)	3.1 (3.5)		3.2 (3.3)	
Score ≥ 8	37 (12.1)		34 (10.9)	
**Survivors**, *n*	368		383	
Age at admission in years, mean (SD)	71.9 (12.0)		71.0 (12.5)	
Female sex	158 (42.9)		150 (39.2)	
Highest education		174		195
Primary school	61 (31.4)		54 (28.7)	
Secondary school	75 (38.7)		76 (40.4)	
University	58 (29.9)		58 (30.9)	
Diagnosis				
Transient ischaemic attack	109 (29.6)		114 (29.8)	
Ischaemic stroke	226 (61.4)		234 (61.1)	
Intracerebral haemorrhage	33 (9.0)		35 (9.1)	
Stroke severity^a^				
NIHSS, median (IQR)	3 (1–5)		3 (1–5)	
Mild (NIHSS ≤ 3)	161 (62.2)		163 (60.6)	
Moderate (NIHSS 4–10)	74 (28.6)		73 (27.1)	
Severe (NIHSS > 10)	24 (9.3)		33 (12.3)	
mRS score, median (IQR)	1 (0–2)	77	1 (0–2)	50
Discharge destination				
Home or rehabilitation	305 (82.9)		329 (85.9)	
Nursing home	51 (13.9)		39 (10.2)	
Other	12 (3.3)		15 (3.9)	
HADS anxiety score		40		33
Mean (SD)	4.9 (4.3)		4.4 (4.2)	
Score ≥ 8	81 (24.7)		77 (22.0)	
HADS depression score		35		28
Mean (SD)	4.4 (4.4)		4.3 (4.1)	
Score ≥ 8	70 (21.0)		80 (22.5)	

Values expressed as *n* (%) unless otherwise stated

*SD* standard deviation, *HADS* Hospital Anxiety and Depression Scale, *NIHSS* National Institutes of Health Stroke Scale, *IQR* interquartile range, *mRS* modified Rankin Scale

^a^Stroke survivors only (ischaemic stroke/intracerebral haemorrhage)

Survivors with non-participating caregivers were on average 3 years younger (*p* ≤ 0.003), and there was some evidence that survivors with non-participating caregivers at 12 months had had slightly milder strokes (when NIHSS was trichotomized into mild, moderate, and severe strokes, *p* = 0.03) (see Online resource 1). For the participating and non-participating survivors (Online resource 2), both groups were balanced on all characteristics except for discharge destination: non-participants were more likely to have been discharged to nursing home (3 months participants 14% vs 20%, *p* = 0.01; 12 months participants 10% vs. 16%, *p* = 0.02).

**Fig. 2:**
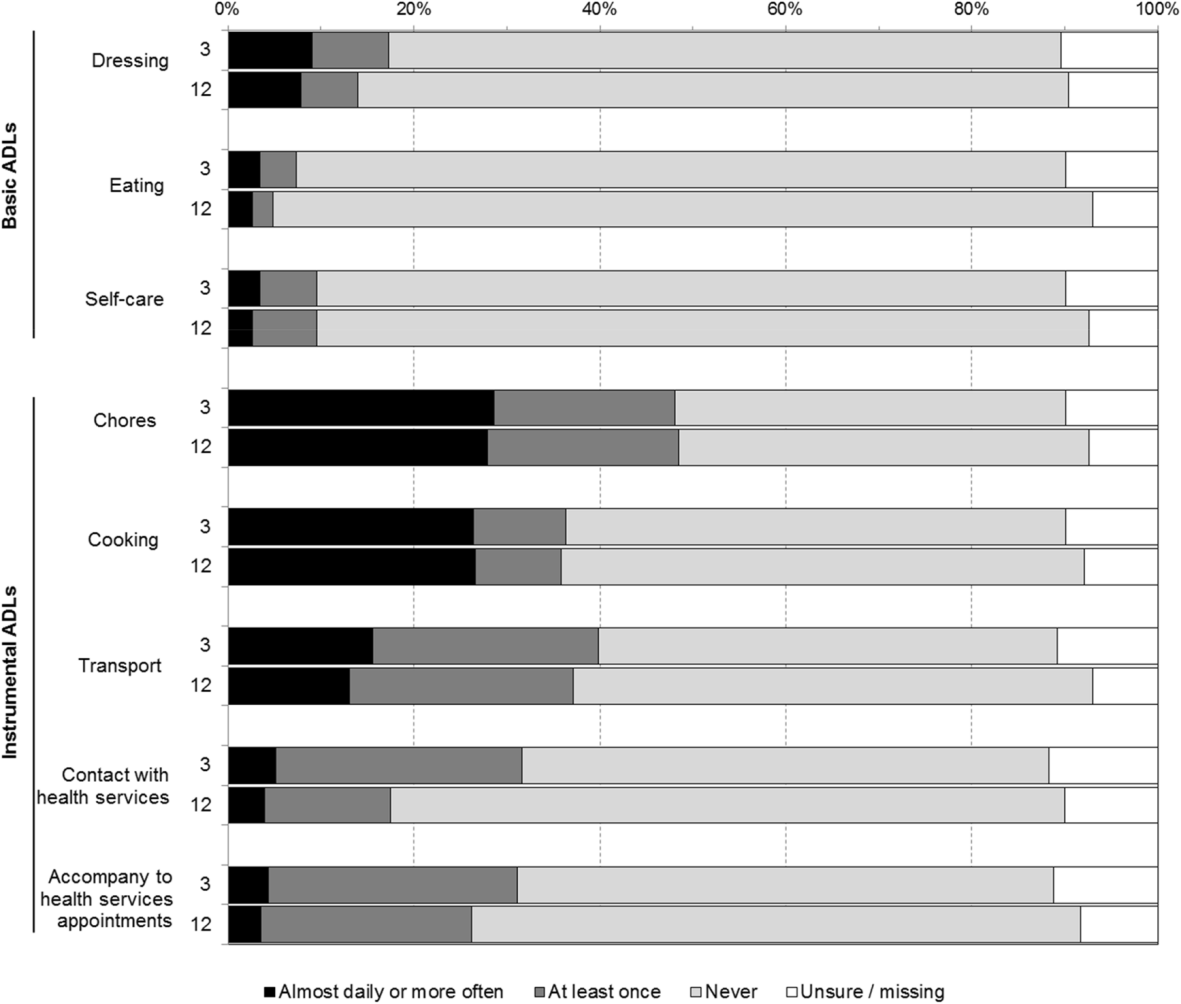
Amount of help given by caregivers of stroke survivors during the last seven days. *ADL* activities of daily living

A larger proportion of caregivers reported that they had to take time off from work in relation to caregiving at 3 months (14%, 17/120) compared to at 12 months (7%, 9/135) (*p* = 0.048). The survivors with stroke primarily received help with instrumental ADLs such as chores (48%), cooking (36%), and transport (40%) (Fig. 2).

### Caregivers’ health-related quality of life and association with stroke severity

Caregivers reported an overall mean health state value of 0.83 at both 3 and 12 months, which was not reduced compared to age- and sex-matched norm values (Table 2). None of the sub-groups analysed (caregivers of survivors with TIA, stroke, different stroke sub-types, and severities) reported reduced HRQoL compared to population norm values at either time point. Caregivers of survivors with mild stroke reported slightly higher health state values than matched norm values (mean caregiver ${\Delta }_{U}$ was + 0.05 at 3 months and + 0.07 at 12 months, both *p* ≤ 0.002), as did caregivers of survivors with ischaemic stroke at 3 months (mean caregiver ${\Delta }_{U}$ +0.03, *p* = 0.03), and caregivers of survivors with ICH at 12 months (mean caregiver ${\Delta }_{U}$ +0.08, *p* = 0.03). Caregiver HRQoL did not change between time points, with the exception of caregivers of survivors with ICH (caregiver ${\Delta }_{U}$ 3 vs. 12 months was + 0.17, *p* = 0.005). The minimal important difference (MID) of EQ-5D-3L health state values is estimated to have a mean of 0.074 [42].

**Table 2:** Caregivers: health-related quality of life (EQ-5D-3L health state values), with comparison to Norwegian population norms

Survivor disease type	3 months (*n* = 320)			12 months (*n* = 326)			${\Delta }_{{\text{U}}_{\text{12m}}}-{\Delta }_{{U}_{\text{3m}}}$^b^
	*n*	Health state value, mean (SD)	${\Delta }_{{U}_{3{\text{m}}}}$, mean^a^	*n*	Health state value, mean (SD)	${\Delta }_{{U}_{\text{12m}}}$, mean^a^	
Transient ischaemic attack	89	0.84 (0.22)	+ 0.04	97	0.82 (0.25)	+ 0.02	− 0.02
Mild stroke (NIHSS ≤ 3)	138	0.85 (0.19)	+ 0.05**	130	0.87 (0.17)	+ 0.07***	+ 0.02
Moderate stroke (NIHSS 4–10)	66	0.79 (0.29)	− 0.02	66	0.80 (0.27)	+ 0.00	+ 0.03
Severe stroke (NIHSS > 10)	27	0.76 (0.32)	− 0.05	33	0.79 (0.32)	− 0.02	+ 0.04
All stroke	231	0.82 (0.24)	+ 0.02	229	0.84 (0.23)	+ 0.04*	+ 0.02
Ischaemic stroke	196	0.83 (0.23)	+ 0.03*	199	0.83 (0.23)	+ 0.03	− 0.01
Intracerebral haemorrhage	35	0.76 (0.31)	− 0.09	30	0.89 (0.20)	+ 0.08*	+ 0.17**
All	320	0.83 (0.23)	+ 0.02	326	0.83 (0.24)	+ 0.03*	+ 0.01

*SD* standard deviation, *NIHSS* National Institutes of Health Stroke Scale

*p < 0.05; **p < 0.01; ***p ≤ 0.001

^a^Mean difference from Norwegian age-sex matched pooled population norms

^b^*t* test for partially overlapping samples

**Fig. 3:**
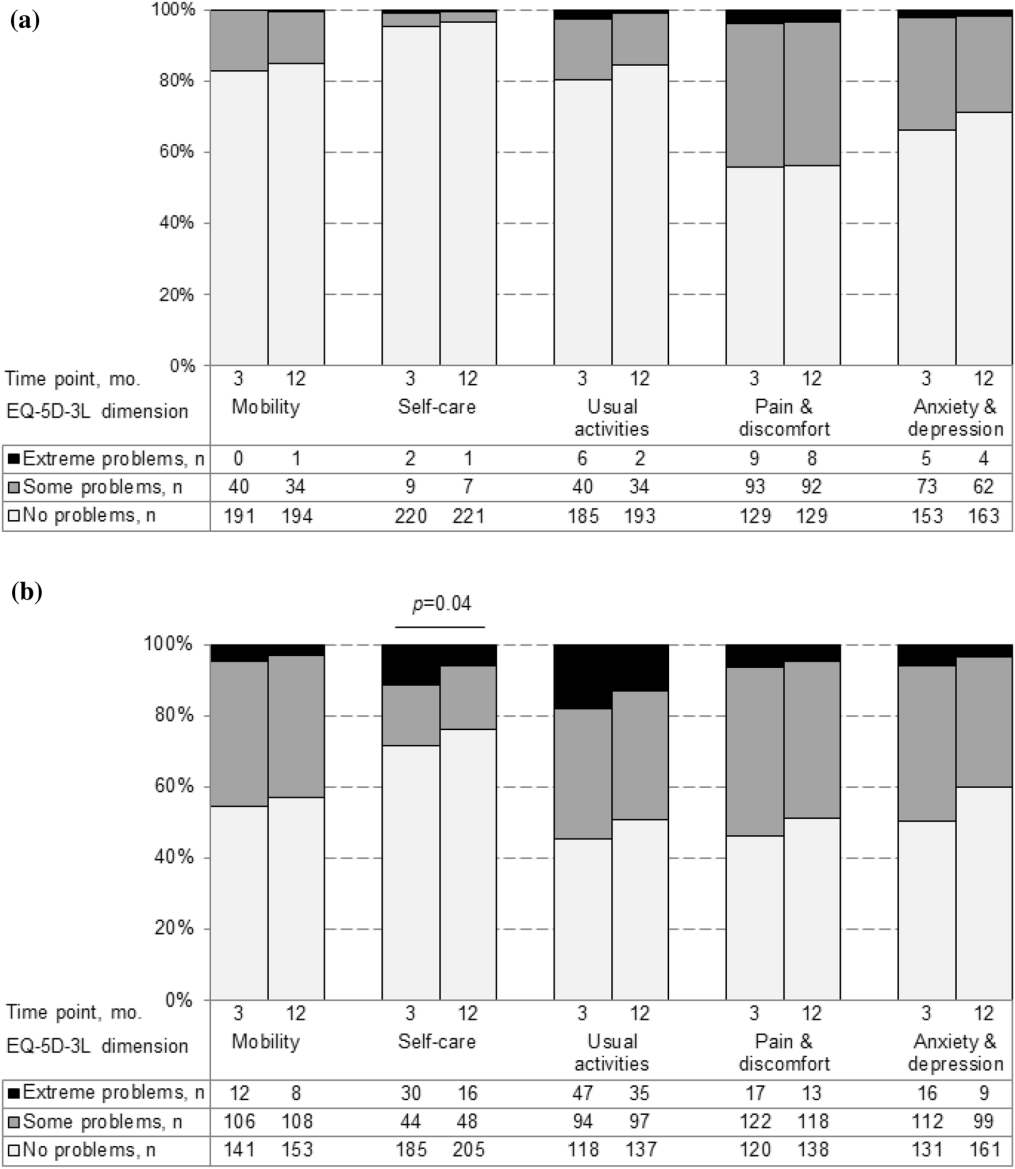
EQ-5D-3L dimensions at 3 and 12 months for **a** the caregivers of stroke survivors, and **b** the stroke survivors

The majority of the caregivers of stroke survivors (Fig. 3) did not report problems with the five health dimensions captured by the EQ-5D-3L. However, at 3 months 44% did report some or extreme problems with pain and discomfort, and 34% with anxiety and depression, with similar proportions at 12 months. There was no evidence for a change in any of the dimensions between 3 and 12 months (all *p* > 0.05).

Mean caregiver health state values decreased incrementally with increasing stroke severity (Table 2). Linear regression (Table 3) of NIHSS scores at admission on caregiver ${\Delta }_{U}$ showed that increasing stroke severity was negatively associated at 3 months (Model 2 coefficient − 0.007, *p* = 0.008). However, when full-adjusted this was attenuated, and at 12 months stroke severity was not significantly associated with caregiver ${\Delta }_{U}$ in any of the models. The survivors’ health state values were strongly, and positively, associated with caregiver ${\Delta }_{U}$ (Model 3, 3 months coefficient 0.117, *p* < 0.01; 12 month coefficient 0.253, *p* < 0.001). The results of Model 4, a sensitivity analysis in the sub-set of participants with complete data, suggest that caregiver depression was also an important determinant.

**Table 3:** Caregivers: multivariable linear regression of association of initial stroke severity on deviation from population norm values of health-related quality of life (caregiver ${\Delta }_{U}$)

	Model 1		Model 2		Model 3 (main model)		Model 4 (sensitivity analysis)	
	${\Delta }_{{U}_{\text{3m}}}$	${\Delta }_{{U}_{\text{12m}}}$	${\Delta }_{{U}_{\text{3m}}}$	${\Delta }_{{U}_{\text{12m}}}$	${\Delta }_{{U}_{\text{3m}}}$	${\Delta }_{{U}_{\text{12m}}}$	${\Delta }_{{U}_{\text{3m}}}$	${\Delta }_{{U}_{\text{12m}}}$
	Coefficient (SE)	Coefficient (SE)	Coefficient (SE)	Coefficient (SE)	Coefficient (SE)	Coefficient (SE)	Coefficient (SE)	Coefficient (SE)
*n*	297	297	297	297	270	268	194	210
Initial stroke severity, NIHSS^a^	− 0.006* (0.003)	− 0.004 (0.002)	− 0.007** (0.003)	− 0.004 (0.002)	− 0.001 (0.003)	− 0.003 (0.003)	0.002 (0.003)	0.001 (0.002)
Caregiver age, years			− 0.002* (0.001)	0.000 (0.001)	− 0.002 (0.001)	0.000 (0.001)	− 0.000 (0.001)	0.002 (0.001)
Caregiver female sex			− 0.043 (0.026)	− 0.029 (0.030)	− 0.058* (0.028)	− 0.038 (0.029)	− 0.017 (0.023)	− 0.021 (0.027)
Caregiver relationship								
Spouse/partner					Ref.	Ref.	Ref.	Ref.
Adult child					0.013 (0.037)	0.024 (0.034)	0.007 (0.029)	0.003 (0.031)
Other					− 0.023 (0.056)	− 0.020 (0.051)	0.027 (0.046)	− 0.009 (0.048)
Discharge diagnosis								
Transient ischaemic attack					Ref.	Ref.	Ref.	Ref.
Ischaemic stroke					0.006 (0.033)	0.053 (0.030)	− 0.028 (0.026)	0.058* (0.027)
Intracerebral haemorrhage					− 0.060 (0.056)	0.119* (0.051)	− 0.077 (0.045)	0.126** (0.047)
Discharge destination								
Home or rehabilitation					Ref.	Ref.	Ref.	Ref.
Nursing home					− 0.028 (0.046)	0.035 (0.020)	− 0.006 (0.039)	− 0.034 (0.047)
Other					− 0.024 (0.033)	0.020 (0.064)	0.030 (0.055)	− 0.044 (0.054)
Survivor health state value^b^					0.117* (0.047)	0.253*** (0.046)	0.164* (0.063)	0.119 (0.065)
Survivor mRS^b^							− 0.001 (0.016)	− 0.001 (0.015)
Caregiver anxiety score^b^							− 0.011* (0.004)	− 0.000 (0.004)
Caregiver depression score^b^							− 0.026*** (0.005)	− 0.037*** (0.005)
Survivor anxiety score^b^							0.003 (0.004)	0.003 (0.004)
Survivor depression score^b^							0.011** (0.004)	− 0.004 (0.005)
*R*^2^	0.022	0.009	0.042	0.013	0.091	0.141	0.398	0.416
Adjusted-*R*^2^	0.019	0.006	0.032	0.003	0.056	0.107	0.347	0.370
df	295	295	293	293	259	257	178	194
AIC	− 58.550	− 22.477	− 60.710	− 19.700	− 50.553	− 86.393	− 176.135	− 164.542

*Ref.* reference category, *NIHSS* National Institutes of Health Stroke Scale, *mRS* modified Rankin Scale, *df* residual degrees of freedom, *AIC* Akaike information criterion

*p < 0.05; **p < 0.01; ***p ≤ 0.001

^a^NIHSS score at admission, survivors with transient ischaemic attack assigned NIHSS = 0

^b^Variable collected at same time point as ${\Delta }_{U}$

### Stroke survivors’ health-related quality of life and association with stroke severity

Survivors with TIA reported the highest HRQoL of the survivors included, and the mean health state values of this group were not significantly different from age- and sex-matched Norwegian norms: 0.84 (SD 0.24) at 3 months, and 0.83 (SD 0.24) at 12 months (Table 4). All other sub-groups of survivors reported lower mean health state values compared to the population norms (e.g. mean survivor ${\Delta }_{U}$ at 3 months: mild stroke − 0.06; moderate stroke − 0.31; severe stroke − 0.48). Most survivors reported improved HRQoL between 3 and 12 months (all stroke: mean ${\Delta }_{U}$ 3 vs. 12 months + 0.06, *p* = 0.002), and especially for ICH (mean ${\Delta }_{U}$ 3 vs. 12 months + 0.16, *p* = 0.01) and moderately severe strokes (mean ${\Delta }_{U}$ 3 vs. 12 months + 0.17, *p* < 0.001). However, all groups of stroke survivors still reported lower mean health state values at 12 months compared to matched population norms (all *p* ≤ 0.03).

**Table 4:** Survivors: health-related quality of life (EQ-5D-3L index values), with comparison to Norwegian population norms

Disease type	3 months (*n* = 368)			12 months (*n* = 383)			${\Delta }_{{U}_{\text{12m }}}-{\Delta }_{{U}_{\text{3m}}}$^b^
	*n*	Health state value, mean (SD)	${\Delta }_{{U}_{\text{3m}}}$, mean^a^	*n*	Health state value, mean (SD)	${\Delta }_{{U}_{\text{12m}}}$, mean^a^	
Transient ischaemic attack	109	0.84 (0.24)	+ 0.03	114	0.83 (0.24)	+ 0.02	− 0.01
Mild stroke (NIHSS ≤ 3)	161	0.74 (0.27)	− 0.06**	163	0.76 (0.26)	− 0.05*	+ 0.02
Moderate stroke (NIHSS 4–10)	74	0.48 (0.42)	− 0.31***	73	0.66 (0.32)	− 0.14***	+ 0.17***
Severe stroke (NIHSS > 10)	24	0.32 (0.41)	− 0.48***	33	0.44 (0.42)	− 0.36***	+ 0.12
All stroke	259	0.63 (0.37)	− 0.17***	269	0.69 (0.32)	− 0.11***	+ 0.06**
Ischaemic stroke	226	0.65 (0.36)	− 0.15***	234	0.70 (0.31)	− 0.10***	+ 0.05*
Intracerebral haemorrhage	33	0.47 (0.38)	− 0.34***	35	0.63 (0.33)	− 0.18**	+ 0.16*
All	368	0.69 (0.35)	− 0.11***	383	0.73 (0.30)	− 0.07***	+ 0.04*

*SD* standard deviation, *NIHSS* National Institutes of Health Stroke Scale

*p < 0.05; **p < 0.01; ***p ≤ 0.001

^a^Mean difference from age-sex matched Norwegian pooled population norms

^b^*t* test for partially overlapping samples

The survivors with stroke reported problems with all five dimensions of the EQ-5D-3L (Fig. 3b). At 3 months, 54.4% reported some or extreme problems with usual activities, 53.7% with pain and discomfort, 49.4% with anxiety and depression, and 45.6% with mobility. Problems with self-care were reported by 28.6% of survivors with stroke, and this was the only dimension that showed improvement over time (*p* = 0.04).

There was evidence of a trend in decreasing EQ-5D-3L health state values with increasing disease severity (Table 4). Linear regression (Table 5) showed that among the survivors, initial NIHSS score was associated with survivor ${\Delta }_{U}$ at both 3 and 12 months, even after adjusting for confounding factors (Model 3, 3 month coefficient − 0.014 and 12 month coefficient − 0.011, both *p* ≤ 0.001). The health state value of the survivor’s caregiver was also significantly associated (both time points *p* ≤ 0.004). Model 4, in the sub-set of participants with complete data, suggests that survivor mRS and anxiety were also important determinants.

**Table 5:** Survivors: multivariable linear regression of association of initial stroke severity on deviation from population norm values of health-related quality of life (survivor ${\Delta }_{U}$)

	Model 1		Model 2		Model 3 (main model)		Model 4 (sensitivity analysis)	
	${\Delta }_{{U}_{\text{3m}}}$	${\Delta }_{{U}_{\text{12m}}}$	${\Delta }_{{U}_{\text{3m}}}$	${\Delta }_{{U}_{\text{12m}}}$	${\Delta }_{{U}_{\text{3m}}}$	${\Delta }_{{U}_{\text{12m}}}$	${\Delta }_{{U}_{\text{3m}}}$	${\Delta }_{{U}_{\text{12m}}}$
	Coefficient (SE)	Coefficient (SE)	Coefficient (SE)	Coefficient (SE)	Coefficient (SE)	Coefficient (SE)	Coefficient (SE)	Coefficient (SE)
*n*	368	383	368	383	314	319	224	247
Initial stroke severity, NIHSS^a^	− 0.032*** (0.004)	− 0.020*** (0.003)	− 0.031*** (0.004)	− 0.019*** (0.003)	− 0.014*** (0.004)	− 0.011*** (0.003)	0.004 (0.003)	− 0.001 (0.003)
Survivor age, years			− 0.003* (0.001)	− 0.002* (0.001)	0.000 (0.001)	− 0.002 (0.001)	0.000 (0.001)	0.001 (0.001)
Survivor female sex			− 0.050 (0.033)	− 0.026 (0.030)	− 0.106** (0.034)	0.011 (0.031)	− 0.017 (0.026)	0.037 (0.025)
Diagnosis								
Transient ischaemic attack					Ref.	Ref.	Ref.	Ref.
Ischaemic stroke					− 0.064 (0.040)	− 0.029 (0.036)	0.000 (0.029)	0.030 (0.029)
Intracerebral haemorrhage					− 0.133* (0.066)	− 0.022 (0.063)	0.001 (0.052)	0.068 (0.050)
Discharge destination								
Home or rehabilitation					Ref.	Ref.	Ref.	Ref.
Nursing home					− 0.426*** (0.051)	− 0.314*** (0.054)	− 0.090 (0.045)	− 0.149** (0.048)
Other					− 0.029 (0.093)	− 0.124 (0.077)	− 0.054 (0,066)	− 0.010 (0.055)
Caregiver health state value^b^					0.205** (0.072)	0.277*** (0.067)	0.244** (0.080)	0.022 (0.069)
Survivor mRS^b^							− 0.150*** (0.015)	− 0.136*** (0.013)
Survivor anxiety score^b^							− 0.021*** (0.004)	− 0.015*** (0.004)
Survivor depression score^b^							− 0.004 (0.005)	− 0.011* (0.005)
Caregiver anxiety score^b^							− 0.001 (0.005)	0.003 (0.004)
Caregiver depression score^b^							0.003 (0.006)	0.006 (0.006)
R^2^	0.175	0.118	0.192	0.131	0.374	0.277	0.720	0.669
Adjusted-*R*^2^	0.173	0.115	0.185	0.124	0.358	0.258	0.703	0.651
df	366	381	364	379	305	310	210	233
AIC	196.507	120.388	193.075	118.673	112.896	64.322	− 122.221	− 136.367

*Ref*. reference category, *NIHSS* National Institutes of Health Stroke Scale, *mRS* modified Rankin Scale, *df* residual degrees of freedom, *AIC* Akaike information criterion

*p < 0.05; **p < 0.01; ***p ≤ 0.001

^a^NIHSS score at admission, survivors with transient ischaemic attack assigned NIHSS = 0

^b^Variable collected at same time point as ${\Delta }_{U}$

### Sensitivity analyses

To check the robustness of our results, we also compared the caregivers’ HRQoL to the population norm values collected by a postal method and by a web-based method separately (see Online resource 3), since the norm values reported via the postal method were slightly higher than those obtained by web-based methods [31], and our data were collected using a postal method. Compared to the population norms obtained from a postal questionnaire, the caregivers’ mean health state values were not significantly different, except for the caregivers of survivors with mild stroke, who reported marginally higher values (3 months + 0.03, *p* = 0.02; 12 months + 0.05, *p* < 0.001). In contrast, compared to web-based population norms, caregiver HRQoL was slightly higher at both time points and for multiple sub-groups.

## Discussion

We have studied the HRQoL of caregivers and survivors with TIA and stroke during the first year post stroke. The caregivers included did not report worse HRQoL compared to age- and sex-matched Norwegians at 3 or 12 months post discharge. There was no change over time except in caregivers of survivors with ICH who reported better HRQoL at 12 months compared to at 3 months. There was weak evidence of an association of the survivors’ stroke severity (NIHSS) with caregiver HRQoL at 3 months, however, the survivors’ HRQoL was a more important determinant. Survivors with TIA reported similar HRQoL compared to population norms. On the other hand, and similar to previous studies [12, 43], the survivors with stroke reported significantly worse HRQoL during the follow-up period. There was a negative association between increasing NIHSS score and survivor HRQoL [43–45] that persisted to 12 months post discharge.

This is one of few studies to report HRQoL in caregivers of, and the survivors with TIA specifically [23, 24]. Our findings suggest that these survivors and their caregivers do not experience worse HRQoL than population age- and sex-matched norms. TIA per definition does not leave persisting neurological impairments, however, may nonetheless give other challenges such as cognitive impairment, depression, and fatigue [23–25]. Additionally, this is one of the first studies to use a stroke-specific measure for associations with caregiver HRQoL. Previous studies have examined this relationship among stroke survivors only, and these have also reported a negative association [43, 45]. Among the caregivers in our study, the association was weak, and was attenuated after adjustments for other relevant factors. One existing study on caregiver HRQoL which does include a stroke-specific measure of survivors’ stroke severity [16], reported an association with the mental component summary scale of the SF-12 instrument, and only on univariate analysis. It therefore appears that other factors are more important determinants of caregiver HRQoL than the initial NIHSS score.

The sensitivity analyses (Model 4) among the sub-set of participants with valid HADS and mRS data were consistent with previous findings of associations with caregiver [3, 16, 17] and survivor HRQoL [43, 45]. The results suggest that current anxiety and depression, and survivor function are perhaps more important determinants of HRQoL than initial NIHSS scores. However, this analysis was only possible in a sub-set of participants, and, furthermore, this information may not be routinely available in clinical practice. We found that the survivors included in this study reported higher mean HADS scores than the caregivers (indicating more problems). However, we note that similar proportions of caregivers and survivors reported anxiety sub-scale scores ≥ 8, indicative of mood disorder [40]. Furthermore, there did not appear to be a change over time in either group of participants (proportions reporting scores ≥ 8 at 3 vs. 12 months *p* > 0.05 for both sub-scales, *z*-test for partially overlapping samples).

While there are many studies on different aspects of caregiver well-being and burden, few existing studies report the health state values of caregivers of stroke survivors for direct comparison to our findings. Compared to those that have been previously reported, the participants in the present study (both survivors and caregivers) appear to have reported higher values. Forster et al. [12] reported mean caregiver health state values at 6 and 12 months of 0.78 and 0.77, respectively, and for stroke patients of 0.44 and 0.46. Cramm et al. [11] measured HRQoL during the acute hospital admission only, and reported a mean caregiver health state value of 0.74 and for patients a mean of 0.49. The participants in the latter study were slightly younger than in the present study, and in both cited studies the survivors appear to be relatively more impaired, with mean BI scores of 12.6 and 11.6, respectively, approximately corresponding to moderate disability [46]. In comparison, the mean NIHSS score in our study was 3, which corresponds to mild strokes.

In comparison, a recent study [24] among patients with either TIA or minor stroke (i.e. NIHSS ≤ 3) reported a mean health state value of 0.84; similar to the TIA group in the present study, but higher than the minor stroke group. That study used the EQ-5D-5L instrument (designed to be more sensitive), and focused on the significant impact of fatigue on HRQoL.

Previous studies are heterogeneous in terms of inclusion criteria, choice of instruments, and methodological and statistical methods [2, 3, 18]. Nonetheless, in contrast to our findings, the majority of existing studies report lower HRQoL in caregivers of patients with stroke compared to controls or population norm values [7, 8, 13, 17, 21]. This may be because the survivors in our study predominately had mild strokes, and caregivers did not report helping to a great extent with ADLs, in particular with more ‘demanding’ basic ADLs such as self-care and eating (only between 2.4% and 3.4% of caregivers reported helping with these tasks almost daily or more often). Some of the caregivers included in our study may therefore more accurately represent family members who are ‘potential future caregivers’ rather than current caregivers. A study among spouses of stroke survivors (but not necessarily caregivers) reported that even seven years after the index stroke, their HRQoL was lower than among spouses of matched controls without stroke [14]. However, it is not reported to what extent the spouses in that study provided informal care.

Interestingly, the comparison of survivor characteristics with participating and non-participating caregivers suggests that our caregiver sample was not biased towards caregivers of the youngest and mildest strokes, despite the relatively high HRQoL reported. In contrast, survivors discharged to nursing homes (who would be expected to have poorer HRQoL) were to a lesser extent represented in our survivor sample.

In this study, we have compared participants’ mean health state values with mean age- and sex-matched values from the Norwegian population. Population norms represent the expected HRQoL for a given age and sex group, with an unknown number of comorbidities, and unknown health status of relatives. It is therefore difficult to isolate the impact on HRQoL due to the burden of caregiving. Our results, however, indicate that the magnitude of loss from full health, as measured by the EQ-5D-3L, is more or less as expected given the caregivers’ age and sex. Ideally, the caregivers’ HRQoL would have been compared to a matched group of individuals with a relative without stroke [13], however, this was not available for this study. It is also noteworthy that the EQ-5D-3L may have not been able to capture more subtle or minor impairments in the participants of our study. Although it is validated in populations of stroke survivors [20, 30], the 5-level version (EQ-5D-5L) might perform better in the caregivers and survivors with TIA, as the five-level version was developed precisely for detecting smaller reductions in HRQoL.

The sensitivity analysis comparing the norm values obtained via a postal method and web-based method separately suggests that there may be systematic differences between the two methods. Since our data are collected by a postal method, we believe that this may be more comparable, possibly reflecting the same self-selection mechanisms at play. Importantly, most of the observed differences—the ${\Delta }_{U}$’s—were smaller than the estimated mean MID of 0.074 [42].

### Strengths and limitations

The strengths of this study include the use of a stroke-specific scale to grade stroke severity and the use of a generic HRQoL tool with the ability to generate health state values for comparison with matched population norms. Survivors and caregivers were recruited prospectively, and the hospital admits stroke patients directly and without prior selection. The hospital’s catchment population is large and diverse, represents around 10% of Norway’s population, and contains both rural and urban areas. However, this is a single-centre study and data were collected via a two-stage postal survey, with a modest response rate. The responder rate for the 3-month questionnaire was 45.9% (389 of 848) for caregivers, and 50.8% (431 of 848) for survivors. The rates for the 12-month questionnaire were 47.1% (404 of 858) for caregivers, and 51.3% (440 of 858) for survivors. Analyses of participants versus non-participants showed that survivors with non-participating caregivers were younger and had milder strokes, and that non-participating survivors were more likely to have been discharged to nursing home. We do not have information about the characteristics of non-participating caregivers. Although the participants and non-participants were well-balanced on most characteristics, it is not inconceivable that the participants represent a more motivated and health-literate group compared to non-participants.

A relatively small number of survivors who were included at 3 months died before the 12-month follow-up and were therefore lost (21 of 368). These individuals most likely represent the sickest and have the lowest HRQoL, and any bias would be that the reported 12-month HRQoL was slightly higher than if these individuals had survived and responded.

This is a descriptive study, and we cannot establish causation. The informal caregivers included in this study reported giving relatively little help with ADLs to the survivors compared to existing studies. Some of the included caregivers might therefore more accurately represent family members of stroke survivors rather than informal caregivers. Differing definitions of informal caregivers remain a challenge when assessing the literature. We have not been able to use repeated measures as not all survivors and caregivers responded at both time points, and it is not certain that the caregivers who did so, were the same individual. We had incomplete data for some variables likely to have an effect on participants’ HRQoL (e.g. anxiety and depression, level of function and cognition), and did not have data on the survivors’ pre-stroke function or the caregivers’ comorbidities, including pre-existing mood disorders. It is also feasible that municipal health and social services may have helped to mitigate reduced HRQoL in our sample, however, we have not analysed this aspect in the present study, and previous studies appear inconclusive [5, 6, 47, 48].

## Conclusion

In this study, informal caregivers and survivors with TIA did not report lower HRQoL compared to age- and sex-matched population norms; further studies with more sensitive instruments able to capture subtle impairments in these groups are warranted. Increasing stroke severity at admission, as measured by the NIHSS, may predict subsequent HRQoL loss among stroke survivors up to 12 months post stroke. Caregiver depression and survivor HRQoL appear to be good predictors of caregiver HRQoL, however, given that these may not be routinely available, the survivors’ admission NIHSS score may help identify caregivers at risk of lowered HRQoL, at least in the initial post stroke period.

## Electronic supplementary material

Below is the link to the electronic supplementary material.

Supplementary file1 (DOCX 20 kb)
