# Supplementary material for: The association of stroke severity with health-related quality of life in survivors of acute cerebrovascular disease and their informal caregivers during the first year post stroke: a survey study
Source: Qual Life Res. 2020 May 9;29(10):2679–93. doi: 10.1007/s11136-020-02516-3 (PMC7561590; doi:10.1007/s11136-020-02516-3)
Supplement: Supplementary file 1 — Supplementary file1 (DOCX 20 kb) [file 11136_2020_2516_MOESM1_ESM.docx]

# ELECTRONIC SUPPLEMENTARY MATERIAL

**Online resource 1.** Comparison of survivor characteristics for participating caregivers versus eligible but non-participating caregivers. Values expressed as *n* (%) unless otherwise stated

|  | **3 months** | | | **12 months** | | |
| --- | --- | --- | --- | --- | --- | --- |
| **Survivor characteristic** | **Caregiver participants**  **(*n*=320)** | **Caregiver non-participants**  **(*n*=696)** | ***p*** | **Caregiver participants**  **(*n*=326)** | **Caregiver non-participants**  **(*n*=607)** | ***p*** |
| Age at admission in years, mean (SD) | 73.0 (11.7) | 70.3 (14.5) | **0.004***^a^* | 72.1 (11.9) | 69.3 (14.3) | **0.003***^a^* |
| Female sex | 146 (45.6) | 296 (42.5) | 0.36*^b^* | 138 (42.3) | 261 (43.0) | 0.84*^b^* |
| Diagnosis: |  |  |  |  |  |  |
| - Transient ischaemic attack | 89 (27.8) | 231 (33.2) |  | 97 (29.8) | 208 (34.3) | 0.38*^b^* |
| - Ischaemic stroke | 196 (61.3) | 408 (58.6) | 0.13*^b^* | 199 (61.0) | 347 (57.2) |  |
| - Intracerebral haemorrhage | 35 (10.9) | 57 (8.2) |  | 30 (9.2) | 52 (8.6) |  |
| Stroke severity: *^d^* |  |  |  |  |  |  |
| - NIHSS, median (IQR) | 3 (1–6) | 3 (1–6) | 0.89*^c^* | 3 (1–6) | 3 (1–5) | 0.15*^c^* |
| - Mild (NIHSS ≤ 3) | 138 (59.7) | 261 (56.1) | 0.66*^b^* | 130 (56.7) | 247 (61.9) | **0.03***^b^* |
| - Moderate (NIHSS 4 – 10) | 66 (28.6) | 143 (30.8) |  | 66 (28.8) | 121 (30.3) |  |
| - Severe (NIHSS > 10) | 27 (11.7) | 61 (13.1) |  | 33 (14.4) | 31 (7.8) |  |
| Discharge destination: *^e^* |  |  |  |  |  |  |
| - Home or rehabilitation | 261 (81.6) | 525 (76.1) | 0.15*^b^* | 279 (85.6) | 478 (79.5) | 0.07*^b^* |
| - Nursing home | 47 (14.7) | 129 (18.7) |  | 34 (10.4) | 91 (15.1) |  |
| - Other | 12 (3.8) | 36 (5.2) |  | 13 (4.0) | 32 (5.3) |  |

*Notes:* SD=standard deviation; NIHSS=National Institutes of Health Stroke Scale; IQR=interquartile range; *^a^ t*-test; *^b^* Chi-squared test; *^c^* Wilcoxon rank-sum test; *^d^* stroke survivors only (ischaemic stroke/intracerebral haemorrhage); *^e^* missing=6 for non-participants at twelve months

**Online resource 2.** Comparison of survivor characteristics for participating survivors versus eligible but non-participating survivors. Values expressed as *n* (%) unless otherwise stated

|  | **3 months** | | | **12 months** | | |
| --- | --- | --- | --- | --- | --- | --- |
| **Survivor characteristic** | **Participants**  **(*n*=368)** | **Non-participants**  **(*n*=648)** | ***p*** | **Participants**  **(*n*=383)** | **Non-participants**  **(*n*=550)** | ***p*** |
| Age at admission in years, mean (SD) | 71.9 (12.0) | 70.8 (14.6) | 0.23*^a^* | 71.0 (12.5) | 69.8 (14.3) | 0.16*^a^* |
| Female sex | 158 (42.9) | 284 (43.8) | 0.78*^b^* | 150 (39.2) | 249 (45.3) | 0.06*^b^* |
| Diagnosis: |  |  |  |  |  |  |
| - Transient ischaemic attack | 109 (29.6) | 211 (32.6) |  | 114 (29.8) | 191 (34.7) | 0.28 |
| - Ischaemic stroke | 226 (61.4) | 378 (58.3) | 0.60*^b^* | 234 (61.1) | 312 (56.7) |  |
| - Intracerebral haemorrhage | 33 (9.0) | 59 (9.1) |  | 35 (9.1) | 47 (8.5) |  |
| Stroke severity: *^d^* |  |  |  |  |  |  |
| - NIHSS, median (IQR) | 3 (1–5) | 3 (1–6) | 0.15*^c^* | 3 (1–5) | 3 (1–6) | 0.83 |
| - Mild (NIHSS ≤ 3) | 161 (62.2) | 238 (54.5) | 0.06*^b^* | 163 (60.6) | 214 (59.6) | 0.21 |
| - Moderate (NIHSS 4 – 10) | 74 (28.6) | 135 (30.9) |  | 73 (27.1) | 114 (31.8) |  |
| - Severe (NIHSS > 10) | 24 (9.3) | 64 (14.6) |  | 33 (12.3) | 31 (8.6) |  |
| Discharge destination: *^e^* |  |  |  |  |  |  |
| - Home or rehabilitation | 305 (82.9) | 481 (74.9) | **0.01***^b^* | 329 (85.9) | 428 (78.7) | **0.02***^b^* |
| - Nursing home | 51 (13.9) | 125 (19.5) |  | 39 (10.2) | 86 (15.8) |  |
| - Other | 12 (3.3) | 36 (5.6) |  | 15 (3.9) | 30 (5.5) |  |

*Notes:* SD=standard deviation; NIHSS=National Institutes of Health Stroke Scale; IQR=interquartile range; *^a^ t*-test; *^b^* Chi-squared test; *^c^* Wilcoxon rank-sum test; *^d^* stroke survivors only (ischaemic stroke/intracerebral haemorrhage); *^e^* missing=6 for non-participants at both three and twelve months

**Online resource 3.** Sensitivity analysis: Comparison of caregivers’ health state values with postal and web population norms separately

| **Survivor disease**  **type** | ***n*** | $\boldsymbol{\Delta}_{\boldsymbol{U}_{\boldsymbol{3}\boldsymbol{m}}}$  **postal norm** *^a^* | $\boldsymbol{\Delta}_{\boldsymbol{U}_{\boldsymbol{3}\boldsymbol{m}}}$  **web norm** *^a^* | ***n*** | $\boldsymbol{\Delta}_{\boldsymbol{U}_{\boldsymbol{12}\boldsymbol{m}}}$  **postal norm** *^a^* | $\boldsymbol{\Delta}_{\boldsymbol{U}_{\boldsymbol{12}\boldsymbol{m}}}$  **web norm** *^a^* |
| --- | --- | --- | --- | --- | --- | --- |
| Transient ischaemic attack | 85 | +0.03 | +0.06* | 86 | +0.01 | +0.03 |
| Mild stroke (NIHSS ≤ 3) | 128 | +0.03* | +0.06** | 119 | +0.05*** | +0.08*** |
| Moderate stroke (NIHSS 4–10) | 59 | -0.03 | -0.02 | 59 | -0.01 | +0.01 |
| Severe stroke (NIHSS >10) | 25 | -0.07 | -0.04 | 33 | -0.03 | -0.01 |
| All stroke | 212 | +0.00 | +0.03 | 211 | +0.02 | +0.05** |
| Ischaemic | 182 | +0.02 | +0.04* | 182 | +0.02 | +0.04* |
| Intracerebral haemorrhage | 30 | -0.10 | -0.08 | 29 | +0.07 | +0.10* |
| All included | 297 | +0.01 | +0.03* | 297 | +0.02 | +0.04** |

*Notes:* NIHSS=National Institutes of Health Stroke Scale; *^a^* mean difference from age-sex matched Norwegian population norms; * p<0.05; ** p<0.01; *** p≤0.001
